# Influences of Maternal Stress during Pregnancy on the Epi/genome: Comparison of Placenta and Umbilical Cord Blood

**DOI:** 10.4172/2167-1044.1000152

**Published:** 2014-02-25

**Authors:** Jia Chen, Qian Li, Alexender Rialdi, Elana Mystal, Jenny Ly, Jackie Finik, Taira Davey, Luca Lambertini, Yoko Nomura

**Affiliations:** 1 Department of Preventive Medicine, Icahn School of Medicine at Mount Sinai, New York, NY, USA; 2 Department of Pediatrics, Icahn School of Medicine at Mount Sinai, New York, NY, USA; 3 Department of Oncological Sciences, Icahn School of Medicine at Mount Sinai, New York, NY, USA; 4 Graduate School, Icahn School of Medicine at Mount Sinai, New York, NY, USA; 5 Department of Psychology, Queens College, CUNY, Flushing, NY, USA; 6 Department of Psychiatry, Icahn School of Medicine at Mount Sinai, New York, NY, USA; 7 William E. Macaulay Honors College (Queens), New York, NY, USA

**Keywords:** Stress, Genetics, Epigenetics, Placenta, Cord blood, HPA-axis, Imprinting

## Abstract

**Background:**

Maternal stress during pregnancy is one of the major adverse environmental factors in utero that is capable of influencing health outcomes of the offspring throughout life. Both genetic and epigenetic processes are susceptible to environmental insults in utero and are potential biomarkers of the experienced environment including maternal stress.

**Methods:**

We profiled expression level of six genes in hypothalamic pituitary adrenal (HPA) axis functioning (HSD11B2, SLC6A4, NR3C1, NR3C2, CRHR1 and CRHR2), two imprinted genes (IGF2 and H19) and one neurodevelopmental gene (EGR1), from 49 pairs of placenta and umbilical cord blood (UCB) samples from a birth cohort. We also assessed global methylation levels by LUminometric Methylation Assay (LUMA) and methylation at the imprinting control region (ICR) of IGF2/H19.

**Results:**

Little correlations between paired placenta and UCB were observed except H19 expression (r = 0.31, P = 0.04) and IGF2/H19 ICR methylation (r = 0.43, P = 0.01); gene expression levels were significantly higher (P < 0.001) in placenta than UCB except CRHR1 and CRHR2, which were unexpressed in placenta. Maternal stress correlated higher levels of HPA genes and lower levels of EGR1 and LUMA, but only in placenta. Positive association between maternal stress and IGF2/H19 ICR methylation was present in both placenta and UCB.

**Conclusions:**

Our findings support the notion that adverse in utero environment, as measured by antenatal maternal stress, depression and anxiety, can be observed in the epi/genome of the relevant tissues, i.e. placenta and UCBs, leading to development of molecular markers for assessing in utero adversities.

## Introduction

Increasing evidence suggests that the period of intrauterine development constitutes one of the most critical periods that can influence risks for neurodevelopmental and mental disease of the offspring throughout life. For example, prenatal exposure to broadly defined stress (e.g., stressful life events, psychological problems) has been linked to long-term neurobehavioral development in the offspring [1,2]. The in utero environment is complex and dynamic, and can encompass psychosocial, socioeconomic, lifestyle, and environmental milieu. Consequently, identifying which of these factors, alone and in combination, influences various aspects of health later in life, including mental health, has been challenging. Genetic as well as epigenetic mechanisms, which can functionally regulate gene expression and thus phenotype, are susceptible to environmental insults particularly at early developmental periods and hence have been suggested as potential biomarkers which can serve as integrated measures of the experienced environment [3–6].

A major challenge of epi/genetic biomarker studies is the fact that these processes are tissue or even cell type specific; thus selecting the right tissue of investigation becomes a key issue of study design. Placenta and umbilical cord blood (UCB) are emerging as promising tissues for epidemiologic investigations of environmental influence on neonatal and childhood health. Placenta is the first complex fetal organ to form during development. Once developed it serves as the source of fetal nutrients, water, gas exchange, excretion, and immune regulation. It shares integrated endocrine control with the brain and may play a vital role in fetal growth and neurodevelopment. Importantly, it can be easily collected in a population study setting, although such repositories are still limited. UCB is another promising source of fetal tissues linking the in utero environment and fetal development because of its richness in stem cells and the relative ease in sample collection. However, despite the fetal origin of both tissues, it is not clear whether they share a similar genetic and epigenetic profile and how they reflect the in utero environment imposed by the mother and experienced by the fetus.

In this study, we examined epi/genetic profile of selected genes from ~ 50 placentas – UCB pairs from a birth cohort study that aimed to study the influence of maternal stress on childhood neurodevelopment. Nine candidate genes that encompass three key biological/physiological pathways related to stress response and brain development were selected, including the HPA axis functioning (HSD11B2, SLC6A4, NR3C1, NR3C2, CRH1R, and CRH2R), genomic imprinting (IGF2 and H19) and neurodevelopment (EGR1). We also assessed the global methylation level by LUminometric Methylation Assay (LUMA) and gene specific methylation at the imprinting control region (ICR) of IGF2/H19. We investigated the comparability of these measurements between placenta and UCB and how they correlated with both self-reported stress and clinically diagnosed depression and anxiety disorders during pregnancy.

## Materials and Methods

### Study population

This study utilizes the first 50 pairs of placenta and UBC collected by the Stress in Pregnancy (SIP) study, an on-going birth cohort study, at Icahn School of Medicine at Mount Sinai. Pregnant women were recruited from the prenatal obstetrics and gynecological (OB/GYN) clinic at Mount Sinai Medical Center, which draws patients from East Harlem and the South Bronx in New York City, where the majority of the residents are low-income ethnic minorities. They were recruited at the 2nd trimester of their pregnancy. Exclusion criteria for participation included HIV infection, maternal psychosis, maternal age <15 years, life-threatening medical complications of the mother, and congenital or chromosomal abnormalities of the fetus. Demographic information, including maternal age, ethnicity, education level, welfare status, marital status, and previous obstetric histories were obtained through self-administered questionnaires during the 2nd trimester and diagnostic outcomes of depression and anxiety disorders among mothers during pregnancy were ascertained by the structured clinical interviews for the DSM-IV Axis I [7] diagnoses in the 3rd trimester. The study was approved by the Institutional Review Boards at Icahn School of Medicine at Mount Sinai and Queens College, City University of New York.

### Placenta and UCB collection

UCBs were collected at birth, prior to the delivery of the placenta. Uncoagulated whole blood was collected in citrated tubes for DNA extraction; PAX tubes (PreAnalytiX – Hombrechtikon, Switzerland) were used for collecting uncoagulated whole blood for RNA extraction. The citrated tubes were mixed, aliquoted and stored at −80°C, while PAXgene tubes (Qiagen – Valencia, CA, USA) were left at room temperature for 2 hours and then stored at −80°C. The placenta biopsies were collected from the 4 quadrants of chorionic villi of the placenta, midway between the umbilical-cord insertion and the placental rim, within 6 hours from the time of delivery. Tissue was extensively washed in cold, (4°C) sterile RNase-free phosphate buffered saline (PBS), blotted in sterile gauze, snap-frozen in liquid nitrogen, and stored in an ultra-freezer at −80°C.

### Stress during pregnancy

Psychosocial Stress and Psychological Symptoms during Pregnancy: Perceived stress scale (PSS-14) [8] assessed mothers’ feelings and thoughts about difficulties and problems during the past month. State-trait anxiety inventory (STAI) evaluated the temporary condition of “state anxiety” and the long-standing quality of “trait anxiety” [9]. Life Experience Interview [10] measured the occurrence of stressful events. Only stressful events perceived as negative in the two pertinent areas for pregnant women, i.e., relationships and health, were examined.

Maternal Diagnostic Outcomes and Functional Impairment during Pregnancy: Clinical status of maternal depression and anxiety during pregnancy were ascertained using the Structured Clinical Interview for DSM-IV Axis I Disorders (SCID-I) [7] by doctoral level trained research clinical interviewers (JL and YN). The clinical interview covered a period between the first prenatal visit and the third trimester. DSM-IV diagnosis of major depressive disorder, dysthymia, adjustment disorder with depressed mood, or depressive disorder not otherwise specified was coded as positive for depression. DSM-IV diagnosis of generalized anxiety disorder, phobia, panic disorder, posttraumatic stress disorder, or obsessive compulsive disorder was coded as positive for anxiety. Both definite and probable cases were included. Global Assessment of Functioning (GAF) scores ascertained by the interviewer was dichotomized at 65, a suggested cut-off scores for functional impairment [11].

### Laboratory analyses

Method of RNA isolation from frozen placenta tissue and UCB has been published previously by our group [12]. Global methytion was assayed by LUMA using PyroMark Q24 (Qiagen) [12]. Gene expression was assessed by qPCR using Light cycler 480 (Roche Applied Science).

The IGF2/H19 ICR methylation assay was assembled by selecting the DNA sequence chr11: 2,021,190 – 2,021,248 (GRCh37/hg19 built) which contains the binding site for the CTCF transcriptional repressor. The selected sequence contains 6 CpG dinucleotides (CpG1 - CpG6). The CpG5 dinucleotide contains a SNP (rs10732516) that disrupts methylation, thus we used the average methylation levels of the remainder five CpG sites to represent the ICR methylation.

Methylation of IGF2/H19 ICR was assayed by bisulfite pyrosequencing using PyroMark Q24 (Qiagen).

### Statistical analyses

The differences between gene expressions/methylation in paired tissues (UCB and placenta) were assessed using paired-samples t-test or the Wilcoxon Signed Rank nonparametric test if the data was non-normally distributed. The relationship of the genes expression/methylation between the paired tissues was evaluated using Pearson’s correlation or Spearman’s correlation for data with normalized or non-normalized distribution, respectively. To explore the maternal factors that might influence the levels of genes expression/methylation in placenta or UCB, General Linear Model (GLM) was used for continuous variables, such as perceived stress, numbers of stressful life events experiences, state- and trait-anxiety and One-way analysis of covariance (ANCOVA) was used for diagnostic outcomes such as maternal depression, anxiety disorders, and functional impairment. The models were adjusted for a priori determined covariates, including marital status, maternal education level, and maternal race. All statistical analysis was performed using RStudio Version 0.97.551 statistical software (RStudio, Inc., 2009–2012). All statistical tests were two-sided, and P < 0.05 was considered statistically significant.

### Characteristics of the study population

Descriptive statistics for population characteristics are presented in Table 1. Out of the 50 participants, one was discarded because of unreliable information provided by the participant on the questionnaire leaving the study population of 49. The mean age of the study participants was 26.8 years and the mean gestational age was 39.0 weeks. Majority of the participants (61%) were self-identified as Hispanic/Latino, 24% as Black, 6% as White, and 4% as Asian. About 40% subjects received education beyond high school, 33% were high-school drop-outs, and 8% were still in high school. Approximately 3/4 of participating women were unmarried. Approximately 14% of women had clinically significant depression, 28% had anxiety disorders, and 60% had functional impairment during pregnancy.

## Results

### Comparison of placenta and UCB

**Figure 1:**
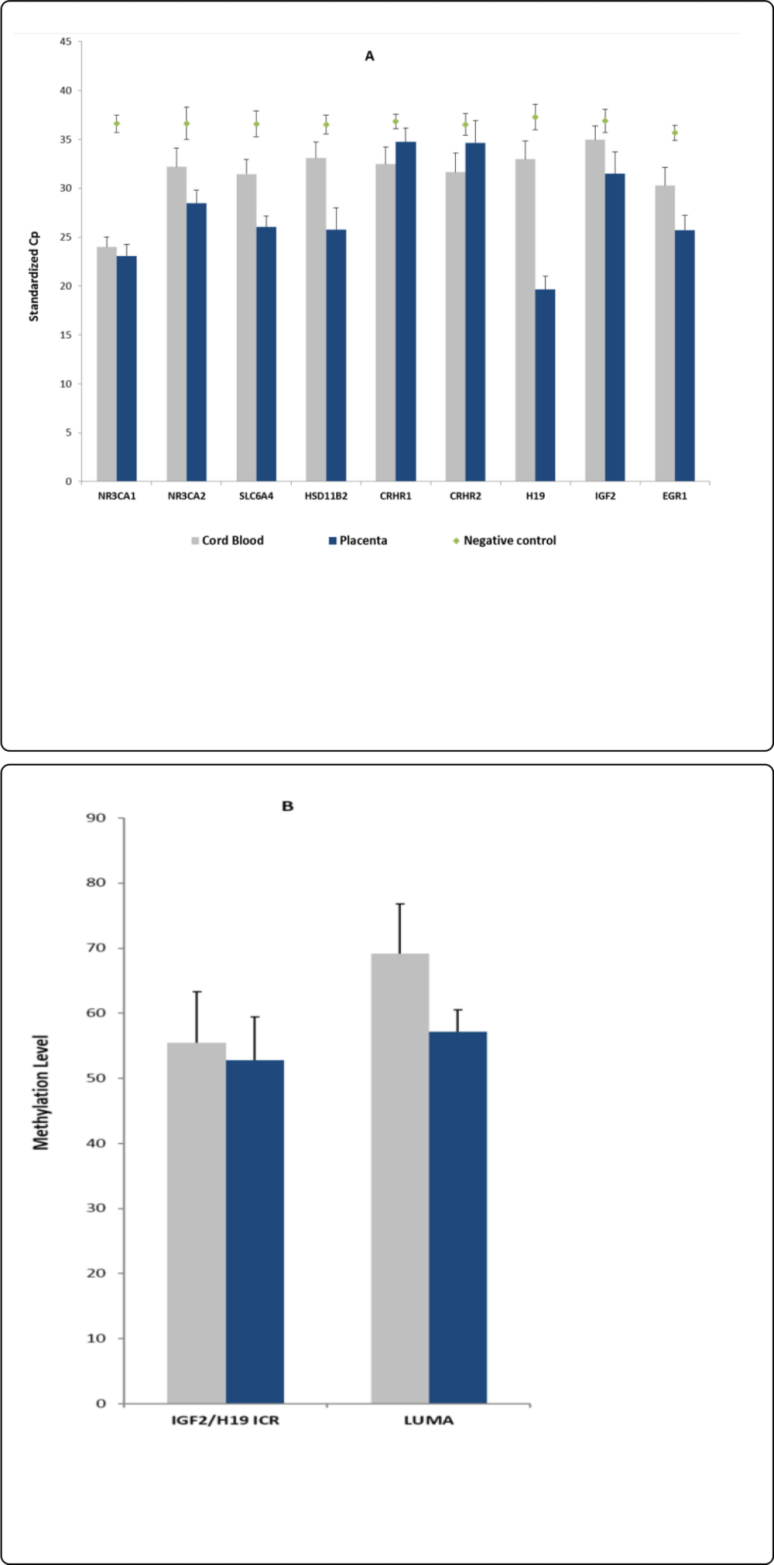
A: Gene expression values between umbilical cord blood and placenta tissue. Cp values measured, standardized, and averaged from all 49 samples. Error bars represent SD. Negative control errors bars represent 3SD. B: Methylation values between cord blood and placenta tissue.

Using qPCR, we quantified mRNA levels from of 9 selected genes: CRHR1, CRHR2, HSD11β2, NR3C1, NR3C2, SLC6A4, EGR1, IGF2, and H19 in 49 paired placenta – UCB samples. The expression levels were standardized to two housekeeping genes – ACTB and 18ssRNA. Figure 1 shows the standardized cycle number (Cp) which inversely related to expression levels, i.e., higher corresponds to lower expression. Values for negative controls (amplification without template) were also illustrated and the values were in the range of 35–37. The figure demonstrates that the tested genes display a broad range of expression in both placenta and UCB tissues, with Cp varying more than 10 reflecting more than 1000 fold difference. NR3C1 was highly expressed in both placenta and UCB. In general, gene expression levels were significantly higher (P < 0.001) in placentas than UCB in 7 out of the 9 tested genes except CRHR1 and CRHR2, which appeared unexpressed in placenta as their Cp values were indistinguishable from those of the negative controls. We observed little correlation between placenta and UCB except H19 which showed moderate correlation (r = 0.31, P = 0.04). H19 was highly expressed in placenta (Cp = 19.64) as compared to UCB (Cp = 32.99); the difference in Cp values translates to over 8000 fold difference between the two tissues (Supplemental Table 1).

We also measured the methylation status of the imprinting control region (ICR) of IGF2/H19. This control region consists of 6 differentially methylated CpGdinucleotides, which are known to bind a transcriptional repressor CTCF protein. Because a C > T single nucleotide polymorphism (SNP) is present on CpG 5 which abolishes the methylation site, we used the average of the remaining 5 CpG sites as the overall methylation level for the ICR. The ICR methylation level was slightly lower in the placenta than that of the UCB (52.81% vs. 55.45%, P = 0.047) with moderate correlation between the two tissues (r = 0.43, P = 0.01) (Supplemental Table 1). For global methylation levels measured by LUMA, UCB appears to have significantly higher methylation levels than placenta (69.13% vs. 57.11, P < 0.001) with no apparent correlations between the two tissues (r = −0.20; P = 0.17) (Supplemental Table 1).

### Antenatal Maternal Stress and Epi/Genetics in Placenta and UCB

We first examined the association between maternal stress during pregnancy and expression levels of candidate genes in both placenta and UCB tissues. Maternal stress was assessed in two broad categories, self-report, and clinical diagnoses (depression and anxiety). Interestingly, the influence of antenatal maternal stress on gene expression was only observed in placenta tissues. Up-regulation of SLC6A4 and HSD11B2 were associated with prenatal perceived stress as well as stressful life events in areas of health-related problems (i.e., negative health-related stress). Enhanced expression of CRHR2 was also associated with antenatal maternal perceived stress. Clinically diagnosed impairment scores were inversely correlated with EGR1 expression (Table 2a).

The influence of antenatal maternal stress on the IGF2/H19 ICR methylation can be observed in both placenta and UCB tissues. While both partner- and health-related stress life events were associated with ICR hypermethylation in the placenta, perceived stress, state anxiety and trait anxiety were associated with ICR hypermethylation in the UCB. Moreover, clinically diagnosed depression and anxiety disorders were associated with decreased global methylation, measured by LUMA, only in the placenta (Table 2b).

## Discussion

Both animal and human studies have demonstrated that prenatal stress affects neurodevelopment in offspring [1,2]. Specifically, prenatally stressed animals have higher basal blood glucocorticoid levels and a reduced number of glucocorticoid receptors in the hippocampus. In humans, prenatal stress adds both physiological and psychological risks for problems in health, cognition, and behavior later in childhood [13]. Even with the best instruments and biomarkers of exposure, exposure assessment, particularly in the context of the developmental origins of health and disease, is difficult to obtain and prone to misclassification and error. The evolving lifestyle, potential variation in the external environment, and most importantly, as psychosocial and perceived stress throughout pregnancy can be experienced by the fetus indirectly through changes in the intrauterine environment and thus may all lead to downstream effects on childhood development. The complexity of exposure assessment, and the encompassing nature of the environment which could impact infant development, provides an impetus to define novel molecular markers. These markers can serve as integrated measures of these various signals and potentially circumvent some of the need to accurately quantify the exposures. We herein explore the hypothesis that the maternal stress, broadly defined, during pregnancy can be captured in the epi/genome of the relevant tissues (placenta or UCB), which may lead to the development of a novel biomarker to quantify antenatal stress exposure.

Stress response is characterized by the activation of the hypothalamus-pituitary-adrenal (HPA) axis and the subsequent increase in glucocorticoid secretion. HPA axis activation during pregnancy is an important adaptive and protective response to stress. However, chronic stress process may result in a continuous HPA axis high-response state, leading to increased glucocorticoid levels and functional disorders of the nervous, endocrine, and immune systems of both the pregnant women and their developing fetuses. In humans, depressed and anxious/stressed maternal mood during pregnancy is associated with elevated cortisol and lower levels of serotonin [14–17] as well as greater risk of preterm delivery and reduced birth weights [18–20]. In this study, we used a candidate gene approach encompassing several pathways including the HPA axis functioning, genomic imprinting and neurodevelopment. The HPA genes include NR3C1, NR3C2, CRHR1, CRHR2, HSD11B2, and SLC6A4. The CRHR1 and CRHR2 bind neuropeptides of the corticotropin releasing hormone which is a key HPA regulator. NR3C1 and NR3C2 encode glucocorticoid receptors that regulate glucocorticoid responsiveness. HSD11B2 carries out the conversion of cortisol to cortisone; and SLC6A4 encodes an integral membrane protein that transports the neurotransmitter serotonin from synaptic spaces intopresynaptic neurons. While early life stress has been shown to be associated with HPA axis genes such as SLC6A4 [21], increasing evidence, including our findings here, suggest that the impact of stress on epi/genetic regulation may happen earlier, even in utero. Although our findings of positive associations between maternal stress and HPA axis genes expression in placenta (HSD11B2, SLC6A4 and CRHR2) are consistent with the only published human study which came from a birth cohort in Rhode Island [22], it contradicts results from animal investigations that are dominating the field [23,24]. Specifically, in the aforementioned human study, placental SLC6A4 expression was significantly increased in women with untreated mood disorders and a non-significant increase was also seen with HSD11B2. The inverse relationship between stress and placental HPA axis gene expression often found in preclinical studies used rodent models, such as HSD11B2 in mice [24] and in rats [23]. Such discrepancy may reflect the differences between human and rodents. It is also possible that the nature of induced stress might not be as salient or rare in occurrence in a human population. Previous studies also showed differences with respect to gender [24,25], tissues types (placenta vs. brain [23]), and even different regions of the brains [25,26], indicating the complex nature of stress-gene association. While gaining further understanding on these issues is important, it is beyond the scope of our study due to mainly relatively small sample size. We acknowledge that future studies should inform these potential differential associations by gender, by tissue types, and different regions of the brain activation.

Genomic imprinting refers to silencing of one parental allele which results in monoallelic expression of the gene in a parental specific fashion; thus imprinted genes are functionally haploid, erasing benefits of diploidy at these loci. Genomic imprinting is one of the leading candidates for mediating the influence of the in utero environment on lifelong health [3–6]. IGF2 and H19 are two reciprocally imprinted genes on chromosome 11. While the paternally expressed IGF2 encodes a member of the insulin family of polypeptide growth factors, which are involved in growth and development, the maternally expressed H19 encodes a non-coding RNA, and functions as a tumor suppressor. The imprinting status is controlled by methylation of the ICR. Although we did not observe any associations between stress and expression of IGF2 and H19, increased methylation (hypermethylation) of the IGF2/H19 ICR was associated with antenatal stress reported by the mothers in both placenta and UCB, indicating the broader effects of stress on the epigenome. While the imprinting status of IGF2/H19 has been correlated with various environmental exposures including bisphenol A [27] and smoking [28], the relationship between IGF2/H19 ICR methylation with maternal stress in our findings further supports this epigenetic marker as an environmental sensor.

As with any biomarker studies, the source of biospecimen under study greatly influences the meaningful interpretation of study results. Given that gene expression and its epigenetic control are tissue specific, selection of the right tissue of investigation is a hot topic in research. We set out to address this issue using two tissues of importance in pregnancy, placenta and UCB, from the same individual. Placenta and UCB, both easily obtainable and non-invasive sources DNA/RNA, are common sources for biomarker discovery reflecting the in utero experience in human observational studies. UCB provides access to specific cell-lineages, and may be an attractive source for studies focusing on the impact of environmental exposures on the differentiation potential of stem cell populations, such as neuronal precursors, and on the immune response of cytokines. The placenta produces many pregnancy related hormones, growth factors and neuroendocrine agents in a timely controlled fashion, thereby fulfilling a critical role in proper intrauterine development. We observed little correlation between placenta and UCB except in imprinted genes, H19 expression and IGF2/H19 ICR methylation; this finding supports the notion that genomic imprinting is established early in development, during embryogenesis; thus the imprinting marks are likely to propagate subsequently through multiple tissues/cell types down the developmental lineage. Our finding of stress – gene expression in only placenta but stress – methylation in both placenta and UCS is an interesting one; it may reflect differences both in physiological response to stress as well as in dynamics of the epi/genome under stress. Nevertheless, factors driving tissue distinctions in the epi/genome profile need to be carefully considered in epidemiologic studies. For example, both placenta and UCB are composite tissues consisting of heterogeneous cell-types; minimizing variability in cell-type composition across samples (sampling placenta in a homogeneous region or cell fractionation of UCB) should be considered with best effort to reduce bias.

Lastly, given the number of genetic markers and stress variables examined in this study, one important issue that warrants careful consideration is the issue of “multiple comparison”, which results in an increased likelihood of false positive findings (Type I error). There is continuing debate on whether/when/how multiple comparisons should be taken into account [29]. When evaluating results of molecular epidemiology studies, statistical power and the priority of the tested hypothesis also need to be taken into account, in addition to the magnitude of the p value [30]. In this study, we opt to report the crude p values without adjusting for multiple comparisons for the following reasons. First of all, instead of an agnostic approach, we selected variables a priori, because of their functional relevance to stress. Second, using the gene-stress matrix, our study focuses on the patterns of associations, rather than the magnitude of associations. Lastly, multiple comparison adjustment, such as Bonferroni method, may safeguard false positive findings, but it may increase Type II error (false negative) and reduce sensitivity [31], thus be too conservative for exploratory purpose. Nevertheless, results from our study need to be interpreted with caution and warrant replication in other population studies.

## Conclusion

In summary, our study supports the notion that psychosocial stress, one of the adverse non-genetic, environmental risks that the growing fetus has been exposed to in utero, can be observed in the placental epi/genome. Methylation of IGF2/H19 ICR in UCBs is also reflective of maternal antenatal stress. Given the relative small size of our study, results of stress – gene association were only presented qualitatively; the magnitude of association is less important and needs to be determined or replicated in larger studies. Nevertheless, our results provided novel mechanistic insights into environmental insults in early developmental stages that may set the trajectory of suboptimal development; it may lead to a discovery of useful biomarkers with significant clinical and public health implications, providing an opportunity to develop early targeted diagnostic tools and early interventions for at-risk children.

## Supplementary Material

supplemental table

## Figures and Tables

**Table 1:** Demographic and stress characteristics of the study population (N=49)

**Demographics**	**N (%)**
Mother’s ethnicity	
Black	12 (24%)
Hispanic/Latino	30 (61%)
White	3 (6%)
Asian	2 (4%)
Others	2 (4%)
**Mother’s educational attainment**	
Primary school education	2 (4%)
Some high school/drop-out	16 (33%)
High school graduate or GED	11 (22%)
Some college	10 (20%)
College degree	8 (16%)
Graduate degree	2 (4%)
**Mother’s marital status at delivery**	
Married	11 (22%)
Single	35 (71%)
Divorced/separated	3 (6%)
Maternal age (years), Mean (SD)	26.8 (5.6)
Gestational age at birth (weeks), Mean (SD)	39.0 (2.5)
**Antenatal Stress, Self-Reported**	**Mean (SD)**
Prenatal perceived stress	37.7 (6.4)
State anxiety	38.7 (11.0)
Trait anxiety	38.9 (11.1)
Stress life event - partner (negative)	0.5 (0.9)
Stress life event - health (negative)	0.1 (0.4)
**Antenatal Stress, Clinical Diagnosis**	**N (%)**
Depression a	
No	37 (86%)
Yes	6 (14%)
Anxiety disorder b	
No	31 (72%)
Yes	12 (28%)
Functional Impairment based on GAFc score	
> 65	17 (40%)
=<65	26 (60%)

NB:

a Major depressive disorder, dysthymia, adjustment disorder with depressed mood, or depressive disorder not otherwise specified were coded as positive for depression.

b Generalized anxiety disorder, phobia, panic disorder, post-traumatic stress disorder, or obsessive compulsive disorder were coded as positive for anxiety

c GAF score = general assessment of functioning

**Table 2:** 

a: Influence of maternal stress on gene expression in placenta									
Antenatal stress	NR3 CA1	NR3 CA2	SLC 6A4	HSD 11B2	CR HR1	CR HR2	H1 9	IG F2	EG R1
Self-report *									
Prenatal perceived stress			↑	↑		↑			
State anxiety									
Trait anxiety									
Stress life event - partner (negative)									
Stress life event - health (negative)			↑	↑					
Clinical diagnosis **									
Depression (yes vs. no)									
Anxietydisorder (yes vs. no)									
Impairment score (> 65 vs. =<65)									↓

b: Influence of maternal stress on methylation in cord blood and placenta				
	IGF2/H19 ICR		LUMA	
	Cord blood	Placenta	Cord blood	Placenta
Self-report *				
Prenatal perceived stress	↑			
State anxiety	↑			
Trait anxiety	↑			
Stress life event - partner (negative)		↑		
Stress life event - health (negative)		↑		
Clinical diagnosis **				
Depression (yes vs. no)				↓
Anxiety disorder (yes vs. no)				↓
Impairment score (> 65 vs. =<65)				

* General linear model controlling variables for marital status (married/common law, single, divorced/separated), mother education (less than high school, high-school graduate, college or more than college), and race (Black, Hispanic, and others).

** One-way ANOVA analysis controlling for marital status (married/common law, single, divorced/separated), mother education (less than high school, high-school graduate, college or more than college), and race (black, Latin, and others).

## References

[1] Fujioka T, Fujioka A, Tan N, Chowdhury GM, Mouri H (2001). Mild prenatal stress enhances learning performance in the non-adopted rat offspring. Neuroscience.

[2] Kofman O (2002). The role of prenatal stress in the etiology of developmental behavioural disorders. Neurosci Biobehav Rev.

[3] Wilhelm-Benartzi CS, Houseman EA, Maccani MA, Poage GM, Koestler DC (2012). In utero exposures, infant growth, and DNA methylation of repetitive elements and developmentally related genes in human placenta. Environ Health Perspect.

[4] Banister CE, Koestler DC, Maccani MA, Padbury JF, Houseman EA (2011). Infant growth restriction is associated with distinct patterns of DNA methylation in human placentas. Epigenetics.

[5] Filiberto AC, Maccani MA, Koestler D, Wilhelm-Benartzi C, Avissar-Whiting M (2011). Birthweight is associated with DNA promoter methylation of the glucocorticoid receptor in human placenta. Epigenetics.

[6] Hoyo C, Murphy SK, Jirtle RL (2009). Imprint regulatory elements as epigenetic biosensors of exposure in epidemiological studies. J Epidemiol Community Health.

[7] First MB, Spitzer RL, Gibbins M, Williams J (2002). Structured Clinical Interview for DSM-IV-TR Axis I Disorders, Research Version, Patient Edition With Psychotic Screen (SCID-I/P W/PSY SCREEN).

[8] Cohen S, Kamarck T, Mermelstein R (1983). A global measure of perceived stress. J Health Soc Behav.

[9] Spielberger CD (1989). State-Trait Anxiety Inventory: Bibliography.

[10] Dohrenwend BS, Krasnoff L, Askenasy AR, Dohrenwend BP (2002). The Psychiatric Epidemiology Research Interview Life Events Scale.

[11] Luborsky L (1962). Clinician's judgments of mental health. Arch Gen Psychiatry.

[12] Nomura Y, Lambertini L, Rialdi A, Lee M, Mystal EY (2014). Global methylation in the placenta and umbilical cord blood from pregnancies with maternal gestational diabetes, preeclampsia, and obesity. Reprod Sci.

[13] Ward AJ (1991). Prenatal stress and childhood psychopathology. Child Psychiatry Hum Dev.

[14] Meliska CJ, Martinez LF, Lopez AM, Sorenson DL, Nowakowski S (2013). Antepartum depression severity is increased during seasonally longer nights: relationship to melatonin and cortisol timing and quantity. Chronobiol Int.

[15] Peer M, Soares CN, Levitan RD, Streiner DL, Steiner M (2013). Antenatal depression in a multi-ethnic, community sample of canadian immigrants: psychosocial correlates and hypothalamic-pituitary-adrenal axis function. Can J Psychiatry.

[16] Giesbrecht GF, Campbell T, Letourneau N, Kooistra L, Kaplan B, APrON Study Team (2012). Psychological distress and salivary cortisol covary within persons during pregnancy. Psychoneuroendocrinology.

[17] Doornbos B, Dijck-Brouwer DA, Kema IP, Tanke MA, van Goor SA (2009). The development of peripartum depressive symptoms is associated with gene polymorphisms of MAOA, 5-HTT and COMT. Prog Neuropsychopharmacol Biol Psychiatry.

[18] Gawlik S, Waldeier L, Muller M, Szabo A, Sohn C (2013). Subclinical depressive symptoms during pregnancy and birth outcome--a pilot study in a healthy German sample. Arch Womens Ment Health.

[19] Nasreen HE, Kabir ZN, Forsell Y, Edhborg M (2010). Low birth weight in offspring of women with depressive and anxiety symptoms during pregnancy: results from a population based study in Bangladesh. BMC Public Health.

[20] Sanchez SE, Alva AV, Diez Chang G, Qiu C, Yanez D (2013). Risk of spontaneous preterm birth in relation to maternal exposure to intimate partner violence during pregnancy in Peru. Matern Child Health J.

[21] Gardner KL, Hale MW, Lightman SL, Plotsky PM, Lowry CA (2009). Adverse early life experience and social stress during adulthood interact to increase serotonin transporter mRNA expression. Brain Res.

[22] Ponder KL, Salisbury A, McGonnigal B, Laliberte A, Lester B (2011). Maternal depression and anxiety are associated with altered gene expression in the human placenta without modification by antidepressant use: implications for fetal programming. Dev Psychobiol.

[23] Jensen Peña C, Monk C, Champagne FA (2012). Epigenetic effects of prenatal stress on 11Î^2^-hydroxysteroid dehydrogenase-2 in the placenta and fetal brain. PLoS One.

[24] Pankevich DE, Mueller BR, Brockel B, Bale TL (2009). Prenatal stress programming of offspring feeding behavior and energy balance begins early in pregnancy. Physiol Behav.

[25] Zohar I, Weinstock M (2011). Differential effect of prenatal stress on the expression of corticotrophin-releasing hormone and its receptors in the hypothalamus and amygdala in male and female rats. J Neuroendocrinol.

[26] Greetfeld M, Schmidt MV, Ganea K, Sterlemann V, Liebl C (2009). A single episode of restraint stress regulates central corticotrophin- releasing hormone receptor expression and binding in specific areas of the mouse brain. J Neuroendocrinol.

[27] Susiarjo M, Sasson I, Mesaros C, Bartolomei MS (2013). Bisphenol a exposure disrupts genomic imprinting in the mouse. PLoS Genet.

[28] Murphy SK, Adigun A, Huang Z, Overcash F, Wang F (2012). Gender-specific methylation differences in relation to prenatal exposure to cigarette smoke. Gene.

[29] Michels KB, Rosner BA (1996). Data trawling: to fish or not to fish. Lancet.

[30] Wacholder S, Chanock S, Garcia-Closas M, El Ghormli L, Rothman N (2004). Assessing the probability that a positive report is false: an approach for molecular epidemiology studies. J Natl Cancer Inst.

[31] Rothman KJ (1990). No adjustments are needed for multiple comparisons. Epidemiology.

