# Supplementary material for: Influences of Maternal Stress during Pregnancy on the Epi/genome: Comparison of Placenta and Umbilical Cord Blood
Source: J Depress Anxiety. Author manuscript; Available in PMC 2018 Jun 27. (PMC6020835; doi:10.4172/2167-1044.1000152)
Supplement: supplemental table [file NIHMS965602-supplement-supplemental_table.docx]

| Supplemental table 1. Measureable comparisons between placenta tissue and umbilical cord blood | | | | | | | | | | |
| --- | --- | --- | --- | --- | --- | --- | --- | --- | --- | --- |
|  | | | | | | | | | | |
| Epi/genetics | N | Cord blood | |  | Placenta | |  | *P*-value for ttest * | Correlation * | |
|  |  | Mean | SD |  | Mean | SD |  |  | *r* | *P*-value |
| Gene expression |  |  |  |  |  |  |  |  |  |  |
| NR3CA1 | 43 | 24.00 | 1.03 |  | 23.09 | 1.15 |  | <0.001 | 0.08 | 0.62 |
| NR3CA2 | 43 | 32.19 | 1.91 |  | 28.47 | 1.37 |  | <0.001 | 0.07 | 0.64 |
| SLC6A4 | 43 | 31.46 | 1.52 |  | 26.02 | 1.15 |  | <0.001 | -0.16 | 0.32 |
| HSD11B2 | 41 | 33.09 | 1.62 |  | 25.78 | 2.22 |  | <0.001 | -0.24 | 0.12 |
| CRHR1 | 39 | 32.47 | 1.75 |  | 34.75 | 1.38 |  | <0.001 | 0.01 | 0.97 |
| CRHR2 | 40 | 31.64 | 1.96 |  | 34.65 | 2.27 |  | <0.001 | 0.09 | 0.59 |
| H19 | 43 | 32.99 | 1.82 |  | 19.64 | 1.35 |  | <0.001 | 0.31 | 0.04 |
| IGF2 | 43 | 34.95 | 1.42 |  | 31.48 | 2.25 |  | <0.001 | -0.03 | 0.84 |
| EGR1 | 43 | 30.27 | 1.88 |  | 25.74 | 1.51 |  | <0.001 | -0.13 | 0.40 |
| Methylation |  |  |  |  |  |  |  |  |  |  |
| IGF2/H19 ICR | 37 | 55.45 | 7.89 |  | 52.81 | 6.62 |  | 0.047 | 0.43 | 0.01 |
| LUMA | 49 | 69.13 | 7.64 |  | 57.11 | 3.38 |  | <0.001 | -0.20 | 0.17 |
| * Paired samples t-test and Pearson correlation coefficient and significant test for the following genes: NR3CA2, HSD11B2, CRHR1, CRHR2, H19, IGF2, EGR1 and IGF2/H19 ICR; Wilcoxon Signed Rand test and Spearman correlation coefficient and significant test for the genes: NR3CA1, SLC6A4 and LUMA due to the non-normal distribution. | | | | | | | | | | |
| SD: standard deviation. | | | | | | | | | | |
